# Role of underappreciated vectors in malaria transmission in an endemic region of Bangladesh-India border

**DOI:** 10.1186/s13071-015-0803-8

**Published:** 2015-04-01

**Authors:** Hasan Mohammad Al-Amin, Rubayet Elahi, Abu Naser Mohon, Mohammad Abdullah Heel Kafi, Sumit Chakma, Jennifer S Lord, Wasif A Khan, Rashidul Haque, Douglas E Norris, Mohammad Shafiul Alam

**Affiliations:** International Centre for Diarrhoeal Disease Research Bangladesh (icddr,b), 68 Shaheed Tajuddin Ahmed Sarani, Mohakhali, Dhaka, 1212 Bangladesh; Department of Biochemistry, Virginia Tech, Blacksburg, VA 24061 USA; Department of Microbiology and Infectious Disease, Cumming School of Medicine, University of Calgary, Alberta, T2N1N4 Canada; Liverpool School of Tropical Medicine, Pembroke Place, Liverpool, L3 5QA UK; Johns Hopkins Malaria Research Institute, Department of Molecular Microbiology and Immunology, Johns Hopkins University Bloomberg School of Public Health, Baltimore, MD 21205 USA

**Keywords:** *Anopheles*, *Plasmodium*, Malaria, Species diversity, Vector, Matiranga, Bangladesh

## Abstract

**Background:**

Despite the efforts of the National Malaria Control Programme, malaria remains as an important public health problem in Bangladesh, particularly in the south-eastern region bordering India. Successful malaria control strategies rely on a detailed understanding of the underlying causes of malaria transmission. Here, an entomological survey was conducted in a malaria endemic area of Bangladesh bordering India to investigate the *Anopheles* mosquito community and assess their *Plasmodium* infection status.

**Methods:**

Monthly entomological collections were undertaken from October 2010 to September 2011 in five villages in the Matiranga sub-district, Khagrachari district in Bangladesh, bordering the Indian State of Tripura. CDC miniature light traps were placed inside houses to collect adult *Anopheles* mosquitoes. Following morphological and molecular identification of the female *Anopheles* mosquitoes collected, they were screened for circumsporozoite proteins (CSP) of *Plasmodium falciparum* (Pf), *Plasmodium vivax*-210 (Pv-210) and *Plasmodium vivax*-247 (Pv-247), by ELISA to determine natural infection rates. Variation in *Anopheles* species composition, relative abundance and *Plasmodium* infection rates were analysed between sampled villages.

**Results:**

A total of 2,027 female *Anopheles* were collected, belonging to 20 species. *Anopheles nivipes* was the most abundant species in our test villages during the peak malaria transmission season, and was observed sympatrically with *An. philippinensis* in the studied area. However, in the dry off-peak season, *An. jeyporiensis* was the most abundant species. Shannon’s diversity index was highest in October (2.12) and evenness was highest in May (0.91). The CSP ELISA positive rate overall was 0.44%. *Anopheles karwari* (n = 2), *An. barbirostris* s.l. (n = 1) and *An. vagus* (n = 1) were recorded positive for Pf. *Anopheles kochi* (n = 1) was positive for Pv-210 while *An. umbrosus* (n = 1), *An. nivipes* (n = 1) and *An. kochi* (n = 1) were positive for Pv-247. A mixed infection of Pf and Pv-247 was detected in *An. barbirostris* s.l..

**Conclusion:**

High diversity of *Anopheles* species was observed in areas close to the international border where species that were underestimated for malaria transmission significantly outnumbered principal vector species and these may play a significantly heightened role in malaria transmission.

**Electronic supplementary material:**

The online version of this article (doi:10.1186/s13071-015-0803-8) contains supplementary material, which is available to authorized users.

## Background

There were an estimated 198 million cases and 584,000 deaths attributed to malaria globally during 2013 [1]. In Bangladesh, malaria represents a major public health concern with approximately 27,000 cases and 15 deaths reported during 2013 [2]. Malaria in Bangladesh is markedly seasonal, where the warm and wet months of May-October define the peak malaria transmission season, and the dry and cooler months of November-April define the off-peak season [3]. Although once widespread throughout the country, malaria is now restricted to 13 districts bordering India and Myanmar [4]. In particular, Khagrachari, Bandarban and Rangamati districts along with Cox’s Bazar districts in the Chittagong Hill Tracts (CHT), account for 80% of malaria cases of the country [5].

Caused predominantly by *Plasmodium falciparum* (Pf), the epidemiology and therefore control of malaria in this region is complicated by high local *Anopheles* vector diversity and the fact that most vectors are members of species complexes. These species include *Anopheles* (*Cellia*) *vagus* Dӧnitz, *An.* (*C.*) *jeyporiensis* James and *An.* (*C.*) *nivipes* Theobald in addition to known major vectors *An.* (*C.*) *baimaii* Sallum & Peyton, *An.* (*C.*) *minimus* complex Theobald, *An.* (*C.*) *philippinensis* Ludlow, *An.* (*C.*) *epiroticus* Linton & Harbach, *An.* (*C.*) *aconitus* Dӧnitz and *An.* (*C.*) *annularis* van der Wulp [6,7].

Topologically, the CHT districts vary from other regions of the country being typified by ranges of forested hills and associated valleys with rivers and small lakes [8] that provide abundant oviposition sites for potential vector species. With the presence of a diverse vector community on both sides of the Bangladesh-India border, vector control interventions in this region are currently reported as inadequate [7-9]. The remote and hilly terrain of the border belt areas makes malaria transmission here harder to interrupt. Moreover, unrestricted movement of people across the border for occupation and trade increase the complexity of transmission. Taken together, this has led to malaria persistence in the villages adjacent to the border [10].

Over the decades, the abundance of different *Anopheles* species throughout the endemic areas of Bangladesh has changed [6-8]. Deforestation and changing agricultural practices in the CHT [11] may explain the altered abundance of *Anopheles* species and their vectorial role [7], once prevalent species have been documented as rare in more recent studies [7,8]. However, only a few data on mosquito diversity and abundance in the hilly border belt areas are available [7,8,12]. Prediction of malaria epidemics and plans for control programmes are greatly dependent on knowledge of vector dynamics and distribution [13] because disease transmission depends on the presence or absence of vector species and their specific behaviours [14]. A better understanding of vector species is necessary for any successful integrated control strategy [15]. This entomological survey was undertaken in a malaria endemic area of CHT, near the Bangladesh-India border to reveal the diversity and abundance of *Anopheles* species over the study period, the variation of *Anopheles* mosquito diversity and abundance among villages and distances from the international border and their *Plasmodium* infection status.

## Methods

### Study area

**Figure 1:**
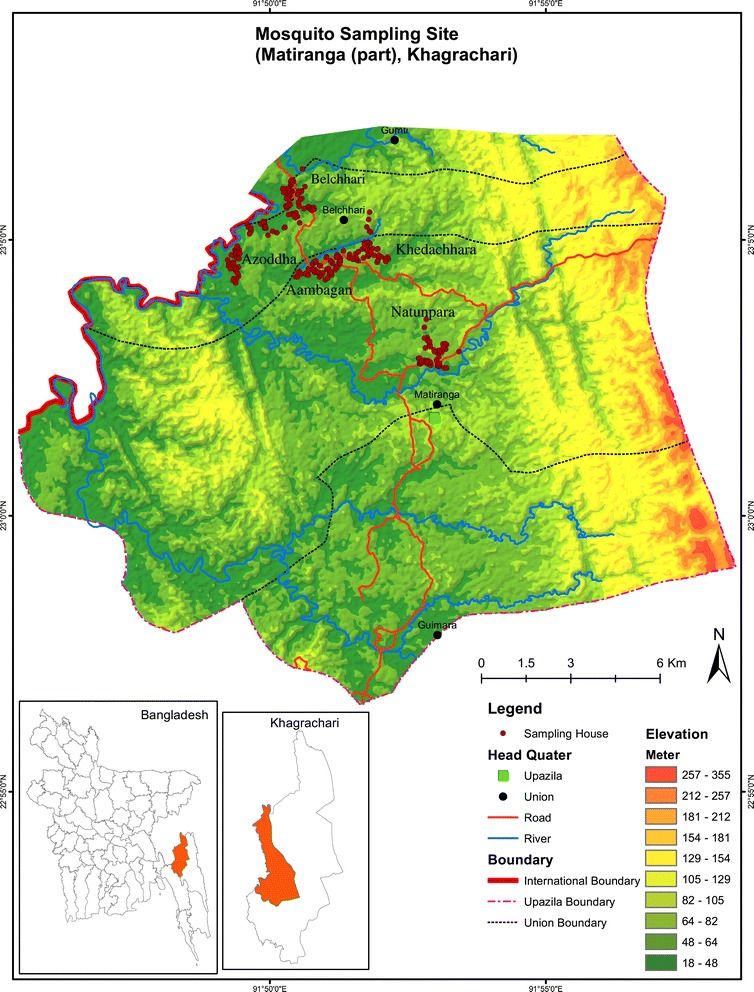
**Map of the study area in Matiranga sub-district, Khagrachari, showing sampled households.**

The study was conducted in the Matiranga sub-district (23°02′19″N, 91°52′36″E) of Khagrachari district, adjacent to the Bangladesh-India border (Figure 1). Matiranga, covers approximately 495 km^2^ and supports 126,477 people at a density of 255.3 per km^2^ [16]. The region has a tropical monsoon climate. During the study period, there was an average rainfall of 217.17 mm and annual average maximum and minimum temperatures of 30.1°C and 20.6°C, respectively and an average humidity of 79.25% (data obtained from a weather station situated at Rangamati which is 15 kilometres away from the study site) were recorded. During surveys, the landscape comprised of paddy fields on the plains and unused shrub lands or teak plantations in hilly areas.

Entomological investigations were conducted in the following five villages: Azoddha, Belchhari, Aambagan, Khedachhara and Natunpara (listed by proximity to the Bangladesh-India international border). Bengali and indigenous people reside in mixed communities, in houses constructed mostly of mud and bamboo. Villages were selected based on high malaria endemicity reports, according to data obtained from the Upazilla Health Complex (UHC) of Matiranga.

### Collection of *Anopheles* mosquitoes

Monthly entomological surveys were undertaken between October 2010 and September 2011 in each village. Adult mosquitoes were collected from the sleeping room of houses using battery operated CDC miniature light traps (Model: 1012, Origin: John W. Hock Inc, USA) between 1800 and 0600 hours. All houses in each village were numbered and an online randomizer (http://www.randomizer.org/form.htm) was used to randomly determine which house to sample.

Each month, five light traps were installed in a village for one night. This enabled five consecutive trap nights in five villages giving 25 traps per month. A total of 60 trap nights were run in each village. Thus, in five villages a total of 300 trap nights were run.

### Processing of mosquitoes

Mosquitoes were taken to the International Centre for Diarrhoeal Disease Research Bangladesh (icddr,b), field laboratory in Matiranga, killed by chloroform and subsequently identified. Mosquito identification to species level was undertaken using a stereomicroscope, according to Peyton *et al.* [17] and Nagpal and Sharma [18]. After identification, each mosquito was preserved in a cryo-vial (labelled with species name, trap number and date) containing desiccant until further analyses.

### Mosquito sample preparation

After field identification, the mosquitoes were taken to the Parasitology Laboratory at icddr,b, Dhaka for further analyses. Each mosquito was dissected into head + thorax and abdomen and homogenized individually in 200 μL and 100 μL of phosphate buffered saline (PBS), respectively. The abdomen homogenate was used for PCR analysis to confirm species identification after DNA extraction as described below and from the head + thorax homogenate 150 μL was used for circumsporozoite protein specific enzyme-linked immunosorbent assay (CSP ELISA).

### Molecular identification of mosquitoes

DNA was extracted from mosquito homogenates using the CHELEX® protocol of Lardeux *et al.* [19] with the following modifications. Briefly, 50 μL CHELEX® -100 (50% v/v) solution was added to 100 μL mosquito homogenate. After an incubation of 15 minutes at 100°C, the mixture was centrifuged at maximum speed for 5 minutes. 30 μL of supernatant was transferred to a new tube and 2 μL Proteinase-K solution was added. After subsequent incubations at 37°C for 45 minutes and at 100°C for 5 minutes the DNA was ready. PCR was undertaken with the extracted mosquito DNA to confirm the taxonomic identification where an established protocol was available. Protocols previously described by Huong *et al.* [20] for confirmation of *An. baimaii*, Phuc *et al.* [21], for species belonging to *An. minimus* complex and the Myzomyia Series, as well as two different protocols of Walton *et al.* [22,23] for confirmation of *An. annularis* and members of the *An.* (*C.*) *maculatus* Theobald group were used. A S1000® Thermal Cycler (Bio-Rad Laboratories, Inc., Hercules, CA, USA) was used for amplification reactions. Post amplification PCR products were electrophoresed on ethidium bromide-stained 1.5% agarose gels, along with Invitrogen® 100 base pair (bp) molecular mass marker (Life Technologies, NY, USA) and visualized under UV illumination.

### CSP ELISA

ELISAs were completed according to Wirtz *et al.* [24] with slight modification (phosphate buffered saline instead of blocking buffer was used to prepare mosquito homogenates) to detect CSP of Pf and two CSP polymorphs of *P. vivax* (Pv-210 and Pv-247). Field caught male mosquitoes were used as negative controls. The positive controls and monoclonal antibodies were obtained from the Centers for Disease Control and Prevention (CDC), Atlanta, USA. The Optical Density (OD) was measured at 410 nm in a Bio-Rad ELISA plate reader, 60 minutes after substrate addition. A cut-off value at least twice the mean OD of the negative controls was considered as positive. All positive samples were confirmed by repeated ELISA.

### Data analysis

Mosquito species diversity and evenness in different months and villages were calculated by using the Shannon’s diversity index (H') and Shannon’s evenness index (E) [25]. Shannon’s diversity index and evenness denote the faunal diversity of a community. Since the sample size of *Plasmodium* positive mosquitoes was too small to allow statistical analyses, mosquito light trap count data of the ten most common *Anopheles* species and whether *Plasmodium* positive was used to compare among villages. The overall variation of mosquito abundance was compared among villages using risk ratio by negative binomial regression because the mean number of mosquitoes is greater than the variance per village. According to Ejercito and Urbino [26] and Nagpal and Sharma [18] the flight range of the ten most common *Anopheles* species collected in this study is generally within one kilometre. Therefore, the distance was taken into consideration to further explore which village shows the maximum variation in terms of mosquito abundance. Villages were compared after adjusting the distance of villages from the international border. Similar analysis was undertaken for each mosquito species to see variation among villages. All data analysis was conducted in R (version 3.0.2) [27]. The geographic positions of the sampled houses were taken with a GPS receiver (Garmin 60CS). ArcGIS 10 (ESRI, CA, USA) was used for map preparation.

### Ethical approval

Written consents were obtained from the households where mosquito collections were conducted. Ethical approval was obtained from Research Review Committee and Ethical Review Committee of icddr,b for this study.

## Results

A total of 2,027 female *Anopheles* mosquitoes were caught (6.76 mosquitoes/trap night, standard error 1.46). Twenty species were confirmed based on taxonomic characteristics and molecular diagnosis for available species complexes. Of 292 mosquitoes morphologically identified as *An. philippinensis*, 247 were molecularly identified as *An. nivipes*, with the reminder (45) being *An. philippinensis*. In the present study, three of 19 specimens morphologically identified as *An. minimus* were molecularly identified as *An.* (*C.*) *varuna* Iyengar. The remaining 16 specimens were confirmed as *An. minimus* (*An. minimus* former species A) by PCR. Of 35 *An. varuna* specimens identified by morphology, one specimen was revised to *An. minimus* after molecular analysis. Concordance between morphological and molecular identification was observed for all other specimens and species.

**Figure 2:**
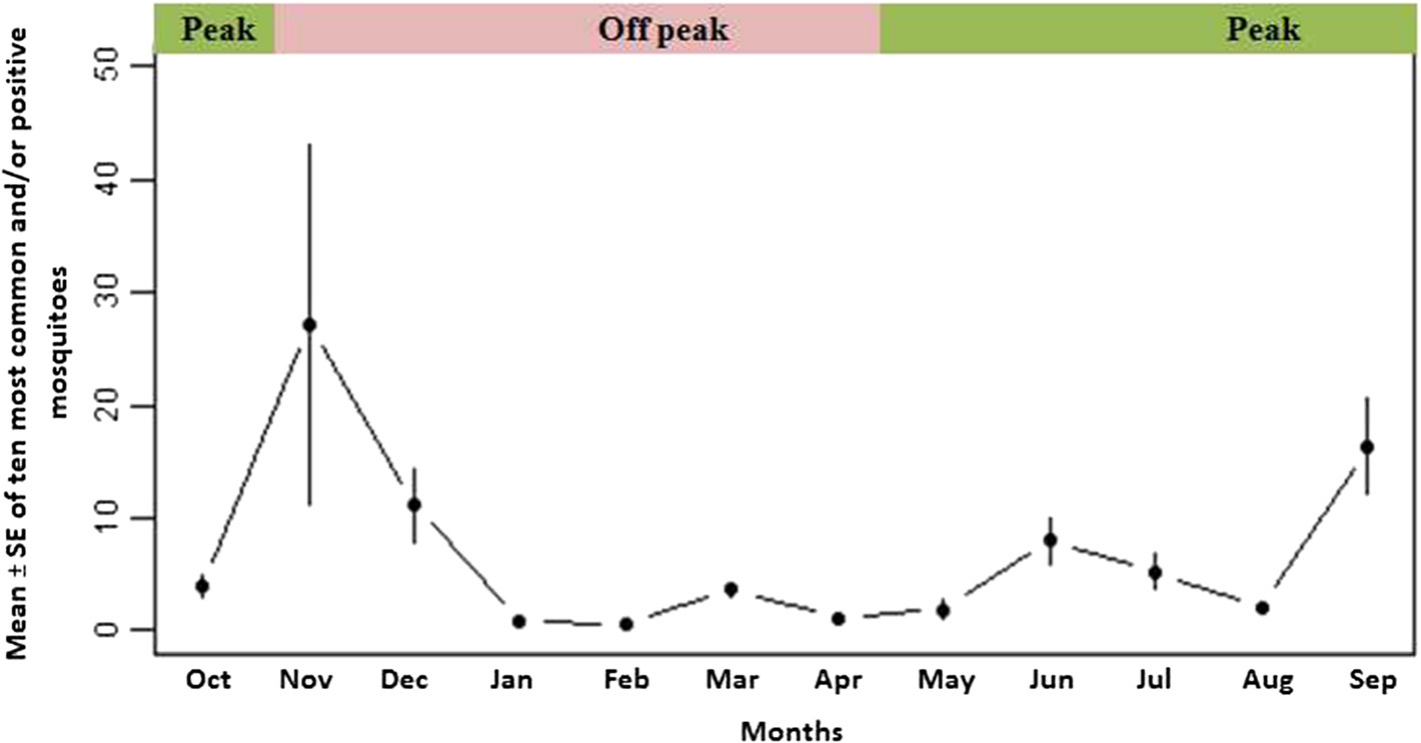
**Temporal distribution of mean ± SE of ten most common and/or positive** ***Anopheles*** **species Matiranga sub-district, Khagrachari.**

*Anopheles jeyporiensis* was observed as the dominant species overall (n = 505, 24.91%), due primarily to a high trap count (n = 267) during November (Table 1 and Figure 2). The next most numerous species were *An. nivipes* (n = 330), *An.* (*C.*) *kochi* Dӧnitz (n = 259), *An.* (*C.*) *karwari* James (n = 219) and *An. vagus* (n = 217) (Table 1). A sharp decrease in the mean number of mosquitoes was observed from January (which is considered as the dry season) to May, the first month of the wet season in Bangladesh. Mean number of mosquitoes began to increase in June (Figure 2). Interestingly, the abundance of traditionally accepted primary vector species such as *An. baimaii*, *An. philippinensis* and *An. minimus* was observed to be much less than newly documented or secondary vector mosquitoes such as *An. jeyporiensis*, *An. vagus* and *An. nivipes* (Table 1).

**Table 1:** **List of** ***Anopheles*** **species collected from five villages (300 trap nights) of Matiranga sub-district, Khagrachari from October 2010 to September 2011**

***Anopheles*** **species**	**Number (%) of** ***Anopheles*** **species in different villages**					**Number**	**(%)**
	**Natunpara**	**Aambagan**	**Khedachhara**	**Belchhari**	**Azoddha**		
Subgenus *Cellia*							
*An. aconitus*	1 (0.31)	3 (1.13)	2 (0.67)	0	7 (0.75)	13	0.64
*An. annularis*	0	0	0	0	1 (0.11)	1	0.05
*An. baimaii*	0	0	3 (1.00)	0	1 (0.11)	4	0.20
*An. jamesii*	0	1 (0.38)	3 (1.00)	0	5 (0.53)	9	0.44
*An. jeyporiensis*	67 (20.81)	73 (27.44)	33 (11.04)	37 (18.05)	295 (31.55)	505	24.91
An. karwari*	45 (13.97)	37 (13.91)	48 (16.05)	24 (11.71)	65 (6.95)	219	10.80
An. kochi*	40 (12.42)	20 (7.52)	18 (6.02)	37 (18.05)	144 (15.40)	259	12.78
*An. maculatus*	0	2 (0.75)	5 (1.67)	1 (0.49)	9 (0.96)	17	0.84
*An. minimus*	0	0	3 (1.00)	0	14 (1.50)	17	0.84
An. nivipes*	29 (9.06)	51 (19.17)	60 (20.07)	52 (25.37)	138 (14.76)	330	16.18
*An. philippinensis*	3 (0.93)	8 (3.01)	7 (2.34)	2 (0.98)	25 (2.67)	45	2.22
*An. subpictus*	2 (0.62)	0	0	0	10 (1.07)	12	0.59
*An. tessellatus*	0	0	0	0	1 (0.11)	1	0.05
An. vagus*	53 (16.46)	26 (9.77)	11 (3.68)	24 (11.71)	103 (11.02)	217	10.71
*An. varuna*	4 (1.24)	3 (1.13)	2 (0.67)	1 (0.49)	27 (2.89)	37	1.83
*An. willmori*	1 (0.31)	0	0	0	1 (0.11)	2	0.10
Subgenus *Anopheles*							
*An. barbirostris* s.l.*	16 (4.97)	11 (4.14)	13 (4.35)	8 (3.90)	45 (4.81)	93	4.59
*An. nigerrimus*	19 (5.90)	4 (1.50)	24 (8.03)	5 (2.44)	8 (0.86)	60	2.97
*An. peditaeniatus*	34 (10.56)	21 (7.89)	61 (20.40)	11 (5.37)	23 (2.46)	150	7.40
An. umbrosus*	8 (2.48)	6 (2.26)	6 (2.01)	3 (1.46)	13 (1.39)	36	1.78
Total	322 (15.88)	266 (13.12)	299 (14.75)	205 (10.11)	935 (46.12)	2027	

*Recorded to be *Plasmodium* positive in the current study.

**Figure 3:**
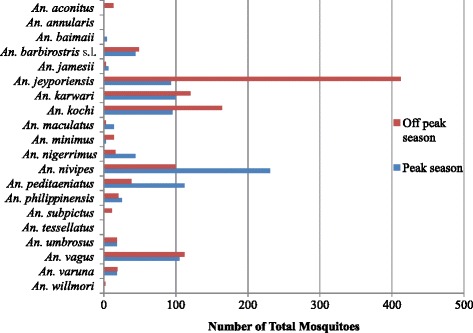
**Relative abundance of** ***Anopheles*** **mosquitoes according to malaria transmission season.**

Species richness was highest (17) in November and lowest (4) in February. However, Shannon’s diversity index was highest in October (H' = 2.12) (Table 2). *Anopheles nivipes* (n = 233) was recorded to be the dominant species during peak malaria transmission season, followed by *An.* (*A.*) *peditaeniatus* Leicester (n = 112), *An. vagus* (n = 105), *An. karwari* (n = 99), *An. kochi* (n = 95), and *An. jeyporiensis* (n = 93) (Figure 3). However, during the off-peak season, *An. jeyporiensis* was the dominant species (n = 412), followed by *An. kochi* (n = 164) and *An. karwari* (n = 120).

**Table 2:** **Shannon’s diversity and evenness indices of collected** ***Anopheles*** **mosquitoes from October 2010 to September 2011**

**Months**	**Species richness**	**Number of mosquitoes**	**Shannon’s diversity index (H')**	**Evenness (E)**
October	13	97	2.12	0.82
November	17	679	1.94	0.68
December	15	277	1.95	0.72
January	5	22	1.34	0.83
February	4	15	1.07	0.77
March	13	90	1.92	0.74
April	8	29	1.52	0.73
May	7	47	1.76	0.91
June	10	183	1.79	0.74
July	11	131	1.78	0.74
August	8	52	1.61	0.77
September	13	405	2.03	0.79

CSP ELISA was carried out on all 2,027 females collected. CSP positivity was observed in nine mosquitoes (0.44%) belonging to six species, which were collected from eight houses (Table 3). *Plasmodium falciparum* infection was reported from four mosquitoes (0.19%): one *An.* (*A.*) *barbirostris* s.l. van der Wulp, one *An. vagus* and two *An. karwari*. A single *An. kochi* was observed to be infected with Pv-210 while three additional mosquitoes (0.15%) identified as *An.* (*A.*) *umbrosus* Theobald, *An. nivipes* and *An. kochi* were positive for Pv-247. Additionally, one *An. barbirostris* s.l. was recorded positive for both Pf and Pv-247.

**Table 3:** **Summary information of CSP ELISA positive** ***Anopheles*** **species and the distance of positive sites from Bangladesh-India international border**

**Mosquito ID**	**Village**	**Species name**	**CSP ELISA**	**Distance (Kilometres)**
KS 0221	Belchhari	*An. karwari*	Pf	1.91
KS 0357	Azoddha	*An. vagus*	Pf	1.27
KS 0515	Azoddha	*An. kochi*	Pv 210	1.23
KS 1004	Aambagan	*An. barbirostris* s.l.	Pf	3.12
KS 1051	Khedachhara	*An. karwari*	Pf	4.54
KS 1147	Azoddha	*An. barbirostris* s.l.	Mixed**	1.2
KS 1154	Azoddha	*An. umbrosus*	Pv 247	1.26
KS 1330	Azoddha*	*An. nivipes*	Pv 247	0.71
KS 1347	Azoddha*	*An. kochi*	Pv 247	0.71

*From same household.

**Mixed = Pf + Pv 247.

The abundance of mosquito species varied among villages. Shannon’s species diversity was highest in Khedachhara (H' = 2.25) and evenness was highest in Natunpara (E = 0.83) (Table 4). Overall mosquito abundance varied significantly (p < 0.05) among the villages while it was significantly greater in Azoddha (p < 0.05) (Additional file 1: Tables S1 and S2). Azoddha was also observed to be significantly (p < 0.05) different from other villages when the distances of villages from the international border was considered (Additional file 1: Tables S3, S4 and S5). In addition, *An. kochi*, *An. vagus*, *An. barbirostris* s.l., *An. nivipes*, *An. peditaeniatus*, *An. jeyporiensis*, *An.* (*A.*) *nigerrimus* Giles and *An. varuna* were significantly (p < 0.05) more abundant in Azoddha (Additional file 1: Tables S6, S7, S8, S9, S10, S11, S12 and S13). Among the eight houses detected harbouring CSP positive mosquitoes, six were located in this village (Table 3).

**Table 4:** **Shannon’s diversity and evenness of** ***Anopheles*** **species in five study villages of Matiranga sub-district, Khagrachari**

**Village**	**Number of mosquitoes collected**	Distance from international border*	**Shannon’s diversity index (H')**	**Evenness (E)**
Natunpara	322	7.8 km	2.18	0.83
Aambagan	266	3.6 km	2.11	0.79
Azoddha	935	1 km	2.14	0.71
Belchhari	205	2.2 km	2.00	0.80
Khedachhara	299	4.7 km	2.25	0.81

*Distance from the international border to the middle point of the village.

## Discussion

Twenty *Anopheles* species were recorded in Matiranga in this study, which is 25% higher than the number of species (n = 15) previously reported from this area during the peak transmission season [8]. Three recent studies in an ecologically and topologically similar region of Bandarban reported 20, 21 and 22 *Anopheles* species, respectively [7,28,12]. Another entomological study in the Tripura state of neighbouring India identified 13 *Anopheles* species during peak transmission in June [9]. The full-year trapping effort may be the reason for the higher species diversity observed in this study. *Anopheles jeyporiensis* was observed to be the dominant mosquito species in the current study. As in a previous study in an ecologically similar area of Bandarban [7], *An. jeyporiensis* (24.91%) was observed to be the dominant mosquito species overall. However, *An. nivipes*, the second most abundant species overall (16.18%) was the most abundant species during the peak transmission season, echoing the findings of Alam *et al.* [7]. Overall, dominancy was observed from *An. vagus* (51.7%) followed by *An. philippine*nsis (22.92%) in another similar study in Bandarban [12].

*Anopheles philippinensis* is considered to be the most important malaria vector in the flood plains of Bangladesh [6]. Recent entomological investigations have reported the abundance of this species in CHT [28,12], as well as in the Matiranga border adjacent to the north-eastern Indian state of Tripura [9]. However, the precise limits of the distribution of this species cannot be defined due to its common misidentification with *An. nivipes*. Alam *et al.* [7] confirmed all specimens morphologically identified as *An. philippinensis* in Bandarban, to be *An. nivipes* by molecular assay. Nagpal and Sharma [29] also stated that previously identified *An. philippinensis* from north eastern India were in fact *An. nivipes*. In the present study, coexistence of *An. nivipes* and *An. philippinensis* was confirmed in Matiranga. One specimen of morphologically identified *An. philippinensis* that was reported positive for Pv-210 in the previous study in Matiranga [8], may have actually been *An. nivipes*. The coexistence of two such closely related species in a malaria hyperendemic area indicates the importance of detailed entomological investigations. As previously reported from the CHT [7], all specimens of *An. minimus* complex collected in the current study were confirmed as *An. minimus* by PCR.

Recent studies have implicated *An. jeyporiensis*, *An. nivipes* and *An. vagus* as potential vectors of malaria parasites in the CHT [7,12]. These three species were similarly abundant in this surveillance study and one specimen each of *An. vagus* and *An. nivipes* were recorded positive for Pf and Pv-247, respectively, confirming their role in malaria transmission in Matiranga. Similar to Bangladesh, these unimportant mosquitoes have also been recorded to be positive for *Plasmodium* in areas of the international borders of Bangladesh. Based on morphological identification, *An. jeyporiensis* was recorded to be positive in a study conducted in Bangladesh-Myanmar border [30]. Molecular evidence had revealed the involvement of *An. nivipes* and *An. vagus* for malaria transmission in north-eastern India, which is adjacent to Matiranga, Khagrachari [31,32].

*Plasmodium* infection in *An. karwari*, *An. kochi* and *An. umbrosus* observed in the present investigation corroborates previous studies [7,8,12]. *Anopheles kochi* has been implicated as a malaria vector of Assam in northeast India [32], and this species and *An. karwari* can be considered as vectors in Bangladesh [7,12]. *Anopheles barbirostris* s.l. was reported to be CSP positive in Sri Lanka for Pf [33], as well as in a previous investigation in Matiranga and Bandarban [8,12]. In the current study, two specimens of *An. barbirostris* s.l. were CSP positive where one specimen was positive for Pf and a mixed infection of Pf and Pv-247 was recorded in the other. Such a mixed infection has not been reported before in *An. barbirostris* s.l. from Bangladesh and is rarely reported in southeast Asia [34]. More studies need to be conducted on these species in order to define their precise role in malaria transmission.

Forests of the CHT have been converted to unused shrub lands, agricultural lands and human settlements over the last 30 years [11,35]. Deforestation can significantly alter the mosquito community by magnifying vector density and abundance in the deforested areas [36,37]. It is evident from the current study that, principal vector mosquitoes, such as *An. baimaii* and *An. minimus* were noted to be very low in abundance compared to more recently implicated vector species.

Increasing population growth and human encroachment into previously under-developed international border areas has resulted in a higher contact rate between human and vector mosquitoes, increasing malaria parasite transmission [38]. In the current study significantly higher mosquito abundance and more ELISA positive mosquitoes were recorded in Azoddha, which is situated within two kilometres of the Bangladeshi-Indian border. During field surveys this village was observed to be surrounded by rice fields, which made this village different than other villages. Since the flight range of most *Anopheles* species is believed to be approximately one kilometre [26,18], malaria transmission dynamics in trans-border areas might be influenced by such local spatial variation of potential vector mosquitoes observed in this study.

## Conclusion

Similar to other endemic areas of CHT, Matiranga has diverse *Anopheles* fauna. However, low densities of recognized vectors and comparatively greater abundance of previously underappreciated *Anopheles* species may play a significantly heightened role in malaria transmission in areas adjacent to the Bangladesh-India border.

## Additional file

Additional file 1:
**Tables of the results from negative binomial regression.**
