# Supplementary material for: Role of underappreciated vectors in malaria transmission in an endemic region of Bangladesh-India border
Source: Parasit Vectors. 2015 Apr 1;8:195. doi: 10.1186/s13071-015-0803-8 (PMC4416289; doi:10.1186/s13071-015-0803-8)
Supplement: Additional file 1: — Tables of the results from negative binomial regression. [file 13071_2015_803_MOESM1_ESM.docx]

**Supplementary Table 1**

Overall variation of mosquito abundance among villages

| Factor | | Regression coefficient of the parameter village | | P value | | 95% confidence interval | |
| --- | --- | --- | --- | --- | --- | --- | --- |
| Village | -0.16 | | 0.04 | | (-0.31, -0.007) | |  |

**Supplementary Table 2**

Comparison of mosquito abundance within villages

| Village | Risk Ratio | P value | 95% confidence interval |
| --- | --- | --- | --- |
| Azoddha | 2.99 | 0.001 | (1.57, 5.66) |
| Belchhari | 0.68 | 0.255 | (0.36, 1.31) |
| Khedachhara | 1.02 | 0.952 | (0.52, 1.98) |
| Natunpara | 1.05 | 0.866 | (0.55, 2.01) |

Reference Variable: Aambagan

**Supplementary Table 3**

| Factor | | Regression coefficient of the parameter distance | | P value | | 95% confidence interval | |
| --- | --- | --- | --- | --- | --- | --- | --- |
| Distance | -0.09 | | 0.02 | | (-0.17, -0.014) | |  |

**Supplementary Table 4**

Comparison of mosquito abundance within villages

| Distance | Risk Ratio | P value | 95% confidence interval |
| --- | --- | --- | --- |
| 2 – < 4 km | 0.43 | 0.002 | (0.25, 0.73) |
| 4 – < 6 km | 0.51 | 0.029 | (0.28, 0.93) |
| 6 – < 8 km | 0.44 | 0.013 | (0.23, 0.83) |
| => 8 km | 0.69 | 0.464 | (0.26, 1.83) |

Reference Variable: < 2 km

**Supplementary Table 5**

Overall variation of mosquito abundance among villages and different distance categories from the Bangladesh-India international border.

| Village | Risk Ratio | P value | 95% confidence interval |
| --- | --- | --- | --- |
| Azoddha | 21.10 | 0.001 | (3.51, 126.72) |
| Belchhari | 2.02 | 0.219 | (0.65, 6.20) |
| Khedachhara | 0.43 | 0.110 | (0.16, 1.20) |
| Natunpara | 0.02 | 0.032 | (0.01, 0.74) |
| Distance | 2.25 | 0.025 | (1.10, 4.60) |

Reference Variable: Aambagan

**Supplementary Table 6**

Abundance of *An. kochi* among villages and different distance categories from the Bangladesh-India international border.

| Village | Risk Ratio | P value | 95% confidence interval |
| --- | --- | --- | --- |
| Azoddha | 90.01 | 0.004 | (5.47, 162.40) |
| Belchhari | 6.61 | 0.027 | (11.7, 40.2) |
| Khedachhara | 0.26 | 0.05 | (4.42, 1.32) |
| Natunpara | 0.01 | 0.03 | (0.008, 1.85) |
| Distance | 2.97 | 0.02 | (1.01, 9.2) |

Reference Variable: Aambagan

**Supplementary Table 7**

Abundance of *An. vagus* among villages and different distance categories from the Bangladesh-India international border.

| Village | Risk Ratio | P value | 95% confidence interval |
| --- | --- | --- | --- |
| Azoddha | 27.38 | 0.006 | (2.41, 37.9) |
| Belchhari | 2.55 | 0.247 | (0.51, 13.8) |
| Khedachhara | 0.18 | 0.017 | (0.037, 1.30) |
| Natunpara | 0.04 | 0.105 | (0.036, 0.09) |
| Distance | 2.31 | 0.05 | (0.93, 6.61) |

Reference Variable: Aambagan

**Supplementary Table 8**

Abundance of *An. barbirostris* s.l. among villages and different distance categories from the Bangladesh-India international border.

| Village | Risk Ratio | P value | 95% confidence interval |
| --- | --- | --- | --- |
| Azoddha | 39.1 | 0.002 | (8.166, 45.2) |
| Belchhari | 8.49 | 0.050 | (0.74, 13.3) |
| Khedachhara | 0.15 | 0.030 | (0.14, 2.61) |
| Natunpara | 0.0037 | 0.001 | (0.00001, 0.251) |
| Distance | 6.55 | 0.001 | (1.49, 35.5) |

Reference Variable: Aambagan

**Supplementary Table 9**

Abundance of *An. nivipes* among villages and different distance categories from the Bangladesh-India international border.

| Village | Risk Ratio | P value | 95% confidence interval |
| --- | --- | --- | --- |
| Azoddha | 12.06 | 0.05 | (0.91, 17.7) |
| Belchhari | 1.95 | 0.43 | (0.27, 9.5) |
| Khedachhara | 0.63 | 0.48 | (0.30, 2.43) |
| Natunpara | 0.03 | 0.09 | (0.0026, 1.87) |
| Distance | 1.85 | 0.17 | (0.76, 5.2) |

Reference Variable: Aambagan

**Supplementary Table 10**

Abundance of *An. peditaeniatus* among villages and different distance categories from the Bangladesh-India international border.

| Village | Risk Ratio | P value | 95% confidence interval |
| --- | --- | --- | --- |
| Azoddha | 0.69 | 0.03 | (0.07, 5.97) |
| Belchhari | 0.33 | 0.40 | (0.16, 6.88) |
| Khedachhara | 3.35 | 0.21 | (0.346, 3.90) |
| Natunpara | 2.74 | 0.72 | (0.008, 7.83) |
| Distance | 0.86 | 0.82 | (0.13, 5.31) |

Reference Variable: Aambagan

**Supplementary Table 11**

Abundance of *An. jeyporiensis* among villages and different distance categories from the Bangladesh-India international border.

| Village | Risk Ratio | P value | 95% confidence interval |
| --- | --- | --- | --- |
| Azoddha | 82.26 | 0.003 | (4.30, 190.07) |
| Belchhari | 2.97 | 0.17 | (0.41, 23.1) |
| Khedachhara | 0.10 | 0.001 | (0.17, 0.55) |
| Natunpara | 0.002 | 0.003 | (0.0007, 0.049) |
| Distance | 3.63 | 0.004 | (1.13, 13.3) |

Reference Variable: Aambagan

**Supplementary Table 12**

Abundance of *An. nigerrimus* among villages and different distance categories from the Bangladesh-India international border.

| Village | Risk Ratio | P value | 95% confidence interval |
| --- | --- | --- | --- |
| Azoddha | 16.23 | 0.03 | (0.14, 96.5) |
| Belchhari | 17.1 | 0.085 | (0.17, 48.6) |
| Khedachhara | 1.44 | 0.81 | (0.06, 2.57) |
| Natunpara | 0.0026 | 0.10 | (0.0003, 1.90) |
| Distance | 5.75 | 0.03 | (0.04, 14.6) |

Reference Variable: Aambagan

**Supplementary Table 13**

Abundance of *An. varuna* among villages and different distance categories from the Bangladesh-India international border.

| Village | Risk Ratio | P value | 95% confidence interval |
| --- | --- | --- | --- |
| Azoddha | 52.98 | 0.04 | (4.7, 113.8) |
| Belchhari | 0.87 | 0.94 | (0.17, 3.41) |
| Khedachhara | 0.32 | 0.37 | (0.18, 4.43) |
| Natunpara | 0.05 | 0.35 | (0.0014, 11.4) |
| Distance | 2.03 | 0.31 | (0.36, 1.35) |

Reference Variable: Aambagan
